# A Tablet-Based Aphasia Assessment System “STELA”: Feasibility and Validation Study

**DOI:** 10.2196/42219

**Published:** 2023-02-08

**Authors:** Yoko Inamoto, Masahiko Mukaino, Sayuri Imaeda, Manami Sawada, Kumi Satoji, Ayako Nagai, Satoshi Hirano, Hideto Okazaki, Eiichi Saitoh, Shigeru Sonoda, Yohei Otaka

**Affiliations:** 1 Faculty of Rehabilitation School of Health Sciences Fujita Health University Toyoake Japan; 2 Deparment of Rehabilitation Medicine Hokkaido University Hospital Sapporo Japan; 3 Department of Rehabilitation Medicine I School of Medicine Fujita Health University Toyoake Japan; 4 Department of Rehabilitation Fujita Health University Hospital Toyoake Japan; 5 Department of Rehabilitation Fujita Health University Nanakuri Memorial Hospital Tsu Japan; 6 Department of Rehabilitation Medicine II School of Medicine Fujita Health University Tsu Japan

**Keywords:** aphasia, tablet, assessment, validity, internal consistency, psychometric, psychological health, stress, digital mental health intervention, digital health intervention, heuristic evaluation, system usability, auditory comprehension, reading comprehension, naming and sentence formation, repetition, reading aloud, Cronbach α, speech

## Abstract

**Background:**

There is an extensive library of language tests, each with excellent psychometric properties; however, many of the tests available take considerable administration time, possibly bearing psychological strain on patients. The Short and Tailored Evaluation of Language Ability (STELA) is a simplified, tablet-based language ability assessment system developed to address this issue, with a reduced number of items and automated testing process.

**Objective:**

The aim of this paper is to assess the administration time, internal consistency, and validity of the STELA.

**Methods:**

The STELA consists of a tablet app, a microphone, and an input keypad for clinician’s use. The system is designed to assess language ability with 52 questions grouped into 2 comprehension modalities (*auditory comprehension* and *reading comprehension*) and 3 expression modalities (*naming and sentence formation*, *repetition*, and *reading aloud*). Performance in each modality was scored as the correct answer rate (0-100), and overall performance expressed as the sum of modality scores (out of 500 points).

**Results:**

The time taken to complete the STELA was significantly less than the time for the WAB (mean 16.2, SD 9.4 vs mean 149.3, SD 64.1 minutes; *P*<.001). The STELA’s total score was strongly correlated with the WAB Aphasia Quotient (*r*=0.93, *P*<.001), supporting the former’s concurrent validity concerning the WAB, which is a gold-standard aphasia assessment. Strong correlations were also observed at the subscale level; STELA auditory comprehension versus WAB auditory comprehension (*r*=0.75, *P*<.001), STELA repetition versus WAB repetition (*r*=0.96, *P*<.001), STELA naming and sentence formation versus WAB naming and word finding (*r*=0.81, *P*<.001), and the sum of STELA reading comprehension or reading aloud versus WAB reading (*r*=0.82, *P*<.001). Cronbach α obtained for each modality was .862 for auditory comprehension, .872 for reading comprehension, .902 for naming and sentence formation, .787 for repetition, and .892 for reading aloud. Global Cronbach α was .961. The average of the values of item-total correlation to each subscale was 0.61 (SD 0.17).

**Conclusions:**

Our study confirmed significant time reduction in the assessment of language ability and provided evidence for good internal consistency and validity of the STELA tablet-based aphasia assessment system.

## Introduction

While people who sustain brain injury from stroke or cranial trauma have higher survival rates today, remaining disabilities caused by brain damage significantly disturb patients’ daily living. Language ability is one of the important cognitive functions in executing social activities that can be impaired by brain injury. Even if individuals regain the physical ability to perform regular activities of daily living, the impairment in language function often makes it difficult for patients to fully reintegrate into their communities. Language function is fundamental to higher brain function, aiding in communication and underpinning logical thinking, social relationships, self-expression, and even dignity. Early detection and prompt and sufficient rehabilitative interventions specific to deficit severity may help such patients regain their independence and ensure reintegration into society. For this to occur, the timely and accurate assessment of language ability is crucial.

Many standardized tests are widely used for evaluating language dysfunction, such as the Western Aphasia Battery (WAB) and Boston Diagnostic Aphasia Exam in clinical settings. However, these tests take considerable time to be administered. Lengthy assessments can pose difficulties for patients with stroke or cranial trauma with language impairments, who often have trouble with long-term concentration due to effects such as inattention and limited endurance [1,2]. Lengthy assessments can cause considerable stress in patients [3], especially when impairments are severe, as they encounter questions they cannot answer. Research has shown that patients with aphasia are especially prone to stress associated with regular linguistic deficits [4,5]. Increased numbers of “unanswerable” questions exacerbate test anxiety [6]; the resulting stress and loss of self-confidence can impede adequate progress in subsequent rehabilitation activities [7].

Several criteria must be met to evaluate language function in a short period of time and in a treatment-oriented manner. The test should be simplified for quicker completion. Conventional language function assessment tests consist of a significant number of questions. For example, the WAB consists of 225 items. Conversely, screening tests performed in shorter amounts of time have also been developed [8]. They usually consist of a small number of items, and their primary focus is typically to ascertain the presence of aphasia and simply estimate language ability in a single dimension.

Although these screening tools are useful for briefly checking deficits in language ability, they do not provide sufficient information for planning treatment; to apply test findings in therapy, “language” should be deconstructed and analyzed for its components. For example, aphasia is traditionally assessed across several modalities, such as auditory and reading comprehension, word and sentence production, and repetition. Clinicians construct rehabilitation plans for individual segments based on their understanding of patients’ performance in each segment [9]. Thus, each language ability component must be evaluated separately to detect the impaired modality and to perform the modality-specific therapy. However, maximizing both simplicity and accuracy of these assessments presents a difficult task ahead. To realize a simple yet detailed language ability test, the use of computer assessment methodologies may be helpful. It may help shorten the assessment process with regard to several aspects. For example, the use of devices such as computers and tablets allows tasks to be presented on screen in a way that is easily understandable [10]; it helps simplify test administration by partially automating the way tasks are explained, presented, and answered [11]; the scoring system can also be automated to reduce administration time and scoring errors [12]. Such a simplification of the test may reduce the stress of patients linked with the assessment of language ability and the respective lengthy testing [3]. In addition, the ability of the devices to record supplemental, objective measures offers additional information to evaluate patients’ language ability (eg, reaction time, which includes critical information on specific aspects of cognitive ability [13]) and is advantageous in the evaluation of brain functions involving complex processes, such as cognition and speech. In fact, various computer assessments have already been used in the field of cognitive testing [14-17]. However, there are still few studies on the assessment of language ability testing using digital devices. A well-validated computer-based testing for language ability would be a useful tool in clinical practice.

The Short and Tailored Evaluation of Language Ability (STELA) is a newly developed computer-based Japanese language ability assessment system for patients with aphasia, which is compact in number of tasks while having the capacity to assess language ability across multiple components. The purpose of this study was to evaluate the clinical feasibility of the STELA by investigating the time-reduction effect of the STELA compared to the WAB as the gold-standard paper-and-pencil test and its internal consistency and validity in assessing the language ability of patients with aphasia.

## Methods

### Study Design and Settings

This was a prospective methodological study with a repeated-measures design, in which the reliability and validity of the STELA were evaluated. The research was conducted in Fujita Health University Hospital and Nanakuri Memorial Hospital.

### Ethical Considerations

This study complied with the principles of the Declaration of Helsinki and was approved by the Medical Ethics Committee of Fujita Health University. All the participants provided written informed consent prior to participation (HM20-060). For privacy protection, the data were deidentified in the analysis.

### Participants

Study participants included patients from the Fujita Health University Hospital’s Department of Rehabilitation and Nanakuri Memorial Hospital, who were diagnosed with impaired language functioning between July 1, 2020, and December 13, 2020. Participants were diagnosed with aphasia on the basis of the WAB results. Inclusion criteria included being 20 years or older, in healthy condition, and confirmed or suspected to have impaired language function when the informed consent was obtained. Patients were excluded if they had a severe cognitive disorder or disturbed consciousness that compromised their ability to follow the instructions during testing.

### Measurements

#### STELA Assessment System

The STELA is a functional language assessment system developed to rapidly, yet comprehensively, evaluate language ability (Sysnet Co. Ltd.). Consisting of a tablet app, a microphone, and an input keypad (for clinician’s use), the system is designed to assess language ability in 2 comprehension modalities (*Auditory comprehension* and *Reading comprehension*) and 3 expression modalities (*Naming and sentence formation*, *Repetition*, and *Reading aloud*). The test comprised 52 questions (Table 1) grouped by difficulty level (see Multimedia Appendix 1 for system development details). For word-related items, difficulty ratings were based on the word’s frequency in regular use; sentence- and text-related items were dependent on the number of clauses. During testing, patients were presented with one question at a time on the tablet, and they responded by pressing the answer on the tablet’s touch screen. When patients had to respond verbally, their performance was recorded using the aforementioned keypad by a speech therapist. This keypad allows the user to input whether the response is correct or incorrect, whether a hint was provided, and the type of error if incorrect (eg, paraphasia and perseveration); it can also record the patient’s voice, as necessary. Responses for each modality were scored in real time according to a predetermined method; the results were displayed at the test’s conclusion. Performance in each modality was scored on the basis of the correct answer rate, wherein 100 points represents a perfect score; overall performance is expressed as the sum of modality scores (out of 500 points). Reaction time and response patterns were recorded during the session and made available as supplemental data for users’ reference.

**Table 1:** The Short and Tailored Evaluation of Language Ability (STELA).

Variables	Items, n	Raw score
Auditory comprehension	16	32
Reading comprehension	16	32
Naming and sentence formation	10	28
Repetition	5	10
Reading aloud	6	12

#### Assessment of Aphasia

The patients were assessed with both the STELA and the Japanese version of the WAB. The WAB is a standardized test battery for evaluating aphasia, widely used as the gold standard in language rehabilitation [18,19]. The reliability and validity of WAB have been previously shown [20]. WAB measures language ability across the following eight modalities: (1) spontaneous speech; (2) auditory comprehension; (3) repetition; (4) naming and word finding; (5) reading; (6) writing; (7) apraxia; and (8) constructional, visuospatial, and calculation tasks. Performance is scored separately for each modality and globally as the WAB–Aphasia Quotient (WAB-AQ), a weighted composite of the subscale scores. The STELA and the WAB were conducted within 14 days, and the order to apply them was randomized for each patient. Both are conducted by the speech therapist in charge of each patient.

The STELA total (global) score and modality (subscale) scores as well as WAB-AQ and the subscales of WAB were used for analysis. The time required from start to finish (completion time) for both scales was also measured.

### Analysis

Total and group-wise comparisons of the completion time for all the tasks of the WAB and the STELA were conducted using the Wilcoxon signed rank test.

The internal consistency and validity of the STELA were evaluated. The STELA’s internal consistency was evaluated for Cronbach α [21] of each modality and item-total correlations. Cronbach α≥.70 is considered adequate for group comparisons, while α≥.90 is considered optimal for clinical applications [22]. Additionally, item-total correlations, that is, the correlation of individual items with the total score of the scale, were evaluated using Spearman correlation coefficient (rho). Items with item-total correlations less than 0.30 should be regarded inconsistent with the other items [23]. The STELA’s concurrent validity was investigated by conducting correlation analysis with the Japanese version of the WAB. Spearman correlation coefficients were used to quantify the agreement between the two tests on each of The STELA’s modalities and global scores (STELA total score versus WAB-AQ). The correlations between the following pairs of modalities were tested (STELA versus WAB): auditory comprehension versus auditory comprehension, repetition versus repetition, naming and sentence formation versus naming and word finding, and reading comprehension + reading aloud versus reading. Correlation coefficients were interpreted as follows: slight correlation; almost negligible relationships—0.00 to 0.20; low correlation—0.20 to 0.40; moderate correlation—0.40 to 0.70; high correlation, marked relationship—0.70 to 0.90; and very high correlation, very dependable relationship—0.90 to 1.00 [24].

### Sample Size Calculation

The sample size estimated Cronbach α using the Bonett method [25], yielding n=16 given the parameters of α=.05, β=.20, and planning value=0.70. For criterion validity, the sample size required for correlation analysis was calculated using G*power software [26], yielding n=21 given effect size=0.50, α=.05, and β=.20 (2-tailed test). The number of samples was set to 30 considering the possibility of data loss.

## Results

In total, 31 patients participated (n=15, 48% male; n=16, 52% female; mean age 59, SD 13.4 years). Their primary diseases were cerebral infarction (n=12, 39%), cerebral hemorrhage (n=13, 42%), subarachnoid hemorrhage (n=2, 6%), brain tumor (n=4, 13%), and cerebral contusion (n=1, 3%). On average, the STELA was administered 91.5 (SD 128.3) days after the event and 4.5 (SD 3.4) days apart from the WAB. Patients’ global scores were the STELA total score=362.5 (SD 12.2; out of 500) versus WAB-AQ=66.9 (SD 28.5; out of 100). The score of the STELA was not normally distributed (*P*=.003), while that of the WAB-AQ was distributed normally (*P*=.08; Table 2).

**Figure 1:**
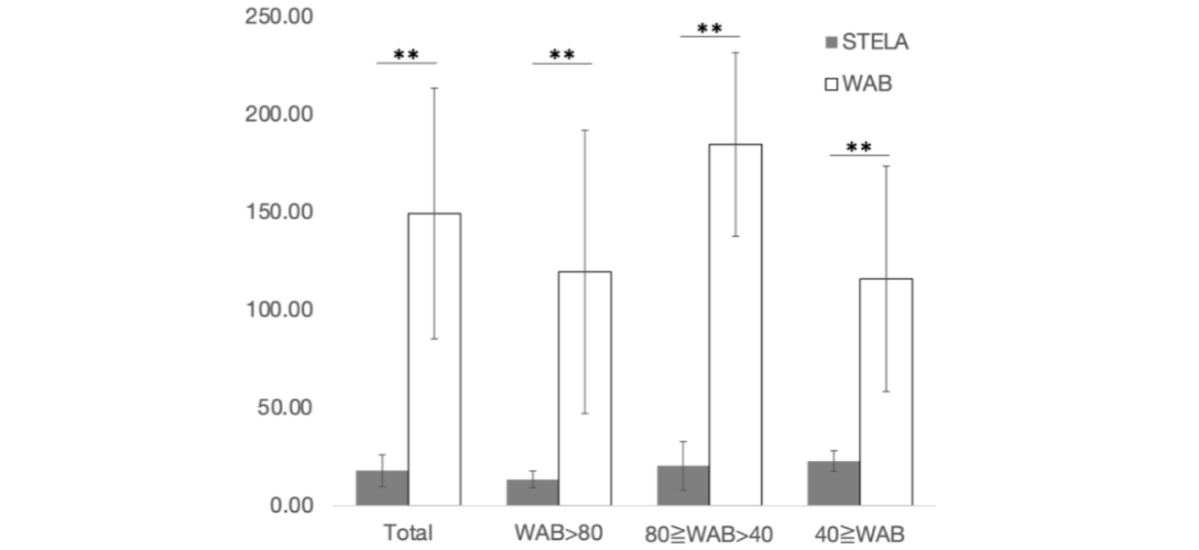
Time taken for testing. Averaged time taken for WAB (white) and STELA (gray) are shown. STELA: Short and Tailored Evaluation of Language Ability; WAB: Western Aphasia Battery. *P<.05, **P<.01.

The time taken to complete the STELA was successfully measured in 27 patients (4 missing values). The time taken to complete the STELA was significantly less than the time for WAB (mean [SD]: 16.2 [9.4] vs 149.3 [64.1] minutes; degree of freedom=26, signed rank statistic (S)=189.0, *P*<.001): this tendency was significant in every WAB category of aphasia severity (WAB-AQ>80: 13.5, SD 4.2 vs 119.5, SD 66.7 minutes, degree of freedom=10, S=33.0, *P*=.001; 80≥WAB-AQ>40: 21.0, SD 10.6 vs 182.7, SD 48.2 minutes, degree of freedom=9, S=27.5, *P*=.002; 0≥WAB-AQ>40: 21.5, SD 5.7 vs 131.7, SD 64.2 minutes, degree of freedom=5, S=10.5, *P*=.03; Figure 1).

The results of the internal consistency evaluation are shown in Table 3. Cronbach α coefficients obtained for each modality were *Auditory comprehension*=.862, *Reading comprehension*=.872, *Naming and sentence formation*=.902, *Repetition*=.787, and *Reading aloud*=.892. Global Cronbach α was calculated as .961. The average of the values of item-total correlation (Spearman rho) to each subscale was 0.61 (0.17). Total item correlations were 0.30 or more for all items and were significant for all but the following four items: 2 items from the word comprehension in *Auditory comprehension*, 1 item from the paragraph comprehension in *Auditory comprehension*, and 1 from the paragraph comprehension in *Reading comprehension*. For each item, Cronbach α was recomputed for each subscale without it—none of the resulting alpha-without-the-item values exceeded the original α by ≥.10.

**Figure 2:**
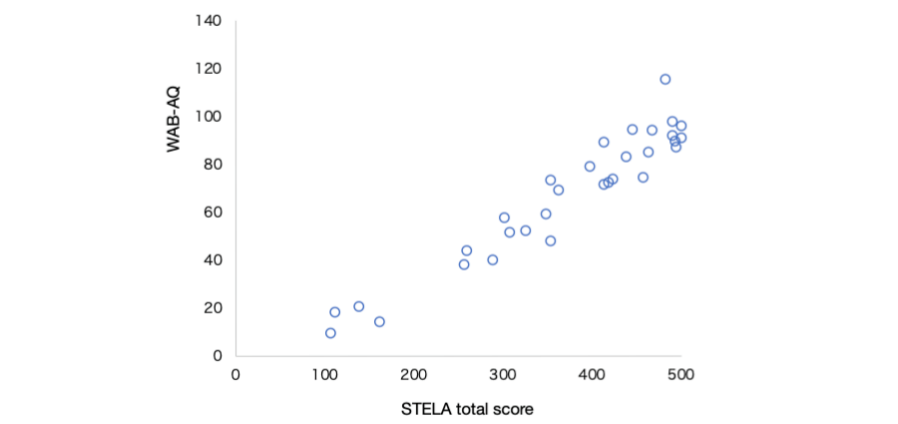
Scatterplots of WAB-AQ and STELA total scores. The correlation coefficient between WAB-AQ and STELA was 0.96 (*P*<.001). STELA: Short and Tailored Evaluation of Language Ability; WAB-AQ: Western Aphasia Battery Aphasia Quotient.

The STELA’s total score strongly correlated with WAB-AQ (ρ=0.93: very high correlation, *P*<.001; Figure 2). Strong correlations were also observed at the subscale level, concerning *auditory comprehension* (ρ=0.75: high correlation, *P*<.001), *repetition* (ρ=0.96: very high correlation, *P*<.001), *naming and sentence formation* (vs WAB naming and word finding:ρ=0.81: high correlation, *P*<.001), and the sum of *reading comprehension* and *reading aloud* (vs WAB reading: ρ=0.82: high correlation, *P*<.001; Table 3).

**Table 2:** Internal consistency.

Modalities				Values			
				Item-total correlation	Alpha without an item		Cronbach alpha^a^
**Comprehension modalities**							
	**Auditory comprehension**						.862
		**Word level**					
			1	0.34	.863		
			2	0.31	.860		
			3	0.50	.851		
			4	0.38	.858		
			5	0.52	.849		
			6	0.45	.857		
		**Sentence level**					
			1	0.56	.849		
			2	0.54	.846		
			3	0.75	.845		
			4	0.31	.864		
			5	0.77	.844		
		**Following commands**					
			1	0.80	.839		
			2	0.80	.848		
		**Paragraph comprehension**					
			1	0.49	.858		
			2	0.52	.862		
			3	0.50	.866		
	**Reading comprehension**						.872
		**Word level**					
			1	0.31	.869		
			2	0.50	.861		
			3	0.45	.868		
			4	0.43	.864		
			5	0.65	.853		
			6	0.50	.861		
			7	0.80	.859		
		**Sentence level**					
			1	0.59	.855		
			2	0.61	.873		
			3	0.48	.869		
			4	0.62	.867		
			5	0.73	.855		
			6	0.56	.859		
		**Paragraph comprehension**					
			1	0.54	.861		
			2	0.56	.873		
			3	0.41	.881		
**Expression modalities**							
	**Naming and sentence formation**						.902
		**Naming**					
			1	0.56	.899		
			2	0.76	.891		
			3	0.73	.888		
			4	0.69	.891		
			5	0.75	.888		
			6	0.69	.895		
		**Picture description**					
			1	0.68	.893		
			2	0.75	.890		
		**Movie description**					
			1	0.92	.895		
			2	0.92	.896		
	**Repetition**						.787
		**Word level**					
			1	0.60	.752		
			2	0.51	.783		
			3	0.51	.783		
		**Sentence level**					
			1	0.94	.673		
			2	0.92	.706		
	**Reading aloud**						.892
		**Word level**					
			1	0.70	.849		
			2	0.69	.865		
			3	0.65	.861		
			4	0.44	.900		
		**Sentence level**					
			1	0.84	.857		
			2	0.96	.895		

^a^Cronbach alpha total=.961.

**Table 3:** Correlations between the Short and Tailored Evaluation of Language Ability (STELA) and the Western Aphasia Battery (WAB) scores.

STELA vs WAB	Spearman correlation coefficient
Auditory comprehension vs auditory comprehension	0.75
Repetition vs repetition	0.96
Naming and sentence formation vs naming and word finding	0.81
Reading comprehension and reading aloud vs reading	0.82
Total vs AQ^a^	0.93

^a^AQ: Aphasia Quotient.

## Discussion

### Principal Findings

This study assessed the clinical feasibility and validity of the STELA, a tablet-based system for evaluating aphasia, by evaluating the administration time, internal consistency, and concurrent validity. The time taken to complete the STELA was significantly less than the time for WAB. The STELA’s total score was strongly correlated with the WAB-AQ, supporting the STELA’s concurrent validity with the WAB as a gold-standard aphasia assessment. Cronbach α coefficients and the values of item-total correlation supported the internal consistency of the STELA.

The STELA took an average of 16 minutes to be administered, approximately one-tenth the duration of WAB, demonstrating a reduced test-taking burden. Long testing sessions are typical of cognitive assessments, including aphasia, causing patients to experience fatigue and stress [3]. Reducing administration time positively influences outcomes by decreasing the time spent in rehabilitation sessions and improving patients’ compliance with training exercises. Given its small question inventory and computerized format, the STELA can be administered in a short period of time; briefness is expected to reduce stress and counteract demotivation for rehabilitation patients.

The STELA’s internal consistency was supported for all modalities and overall, by very high Cronbach α coefficients, measured at .96 for the whole scale and ranging from .79 to .90 for its subscales. Furthermore, all item-total correlations measured in each subscale were .30 or more, and statistically significant except three of them; significance level of correlations for the exceptions were marginal (ie, word comprehension item in *auditory comprehension* and paragraph comprehension item in *auditory comprehension* and *reading comprehension*). Low variation in the response data may be responsible for these exceptions, as the first item of the word comprehension set is the easiest question within the modality, while the third item of the news text comprehension set is the hardest. Nevertheless, no alpha-without-the-item value in any modality (these items included) exceeded the corresponding Cronbach α with the item included by over .10, reflecting good homogeneity in each subscale’s item set.

The STELA’s total score was strongly correlated with WAB-AQ, supporting the former’s concurrent validity concerning a gold-standard aphasia assessment. The stronger the intertest correlations observed at the subscale level, the further support it provides for the STELA’s validity in the corresponding modalities of language function.

Further integration of digital technology can allow the STELA to assess language ability even more rapidly while keeping the granularity. For example, the employment of computer adaptive testing methodology [27] may contribute to further shorten the administration time of the test and reduce the stress of the patients who receive the assessment. Freeing severely impaired patients from distress caused by continually confronting them with challenging questions could be critical to support their motivation to engage in rehabilitation training and adherence. According to the self-determination theory, the feeling of incompetency or lack of control can cause amotivation, which potentially jeopardizes activity adherence [28,29]. Strategies to alleviate the distress caused by an inability to answer questions must be further investigated for language assessments; global assessments wherein difficulty level is adjusted according to impairment severity may help. Nonetheless, applying the approach in a population wherein symptoms vary significantly, such as aphasia, could prove very complicated. Hence, a more in-depth examination of techniques for simplifying tests through digital technology is required.

### Limitations

Since the STELA evaluates language ability using a tablet-based system, in severe cases, patients’ performance could be affected by difficulty in operating the tablet due to concurrent cognitive dysfunction. The system’s scope of application requires further investigation, along with usability concerns (eg, steps to take if patients have trouble using the tablet). Additionally, test-retest reliability was not investigated in this study, as our participants were primarily in the subacute phase after their cerebrovascular event, a period wherein aphasia symptoms can fluctuate significantly in a short period. To evaluate the STELA’s test-retest reliability, a study with a patient population in the chronic phase of illness should be further considered.

### Conclusions

In this study, clinical feasibility of the STELA tablet-based aphasia assessment system was investigated. The results showed the significantly shorter administration time of the STELA compared with that of the WAB as a gold-standard paper-and-pencil test, and the data also supported the internal consistency and the concurrent validity with WAB. These results support the potential usefulness of the STELA in daily rehabilitation practice.

## Appendix

Multimedia Appendix 1 Supplemental methods.

## Abbreviations

S: signed rank statistic
STELA: Short and Tailored Evaluation of Language Ability
WAB: Western Aphasia Battery
WAB-AQ: WAB–Aphasia Quotient

## Data Availability

The data collected and analyzed during the study are available from the corresponding author upon reasonable request.
