# Supplementary material for: A Tablet-Based Aphasia Assessment System “STELA”: Feasibility and Validation Study
Source: JMIR Form Res. 2023 Feb 8;7:e42219. doi: 10.2196/42219 (PMC9947769; doi:10.2196/42219)
Supplement: Multimedia Appendix 1 [file formative_v7i1e42219_app1.docx]

Supplementary Methods

Development of Short and Tailored Evaluation of Language Ability Systematic Test for the Evaluation of Language Ability (STELA)

## Structure of the test

The panel to develop the structure of the test consisted of a group of rehabilitation specialists (a medical doctor and five speech therapists). The panel discussed, 1) which modalities to test, 2) which types of tasks and how many of them to prepare, and 3) how to rate the performance. The modalities to test were selected as follows; firstly, a list of modalities was created from the modalities employed in WAB, BDAE and Standard Language Test of Aphasia (SLTA: most widely used test battery for aphasia in Japan [1],[2]). Then, the panel identified five modalities minimally required for clinical assessment and feasible to assess with the use of tablet, which are Auditory comprehension, Reading comprehension, Naming and sentence formation, Writing Repetition, and Reading aloud. In addition, Writing was also considered important but eliminated from the analysis because the severity of the patients' paresis could affect their performance. Then, types and number of tasks were determined. To make the structure as simple as possible, the panel decided to keep the number of tasks at a minimum. The selected tasks were as follows; Word comprehension, Sentence comprehension, Following commands and Paragraph comprehension for Auditory comprehension; Sentence comprehension and Paragraph comprehension for Reading comprehension; Naming, Picture Description and Movie description for Naming and sentence formation; Word repetition and Sentence repetition for Repetition; Word reading and Sentence reading for Reading aloud. The tasks were prepared in two or three levels, and for each level, one to three tasks were created. The number of tasks and the ratings of Auditory and Reading comprehension were determined to employ multiple selection, while the ratings of Naming and sentence formation, Repetition and Reading aloud were determined to be conducted by the speech therapists with a separate keyboard.

## Word Selection

Words were selected from the vocabulary used in “Act Cards” (ESCOR Ltd.). Picture flashcard sets are often used as language training resources in clinical settings in Japan, wherein words are organized in order of familiarity. The selection criteria and difficulty ratings were determined on the basis of words’ reference data for usage frequency specified in the “Vocabulary and Chinese characters in ninety magazines of today” (National Institute for Japanese Language and Linguistics)[3]. STELA classifies words according to three categories of usage frequency (High: >.114, Mid: .030–.114, Low: <.030). These criteria were determined on the basis of the high-and low-frequency words reported by Fujita et al.[4] (2000). The difficulty was additionally calibrated for words’ semantic category and phonology; for prose-related questions beyond short sentences, clause number and grammatical complexity were additionally considered. Specifically, target and non-target words were chosen from different semantic categories for low-difficulty questions but from the same category for high-difficulty questions. In phonological terms, the target words selected were those that have two-three *morae* of non-special syllables (i.e., no palatalized consonants, geminate consonants, moraic nasals, or long vowels) and lack homophones of the same accent pattern or pronunciation. Non-target words were selected to have distinct phonological features from target words (e.g., different word-initial and word-final sounds). Production questions were ordered to avoid provoking presentation errors; thus, each target word’s initial sound was different from that of the preceding item. To facilitate modality-wise comparisons, the same target words were tested in questions in STELA’s *Naming and sentence formation speech* (Naming), *Repetition* (Word repetition), and *Reading aloud* (Word) components.

1. SLTA committee. Standardezed Language Test of Aphasia: manual of directions. 2nd ed. Tokyo: Homeido; 1977 [Book in Japanese]

2. Isomura-Motoki, A., and Mimura, M. Neuropsychology in Japan. In *The neuropsychology of Asian Americans,* 249-258. Psychology Press.

3. Institute NLR. Vocabulary and Chinese Characters in Ninety Magazines of Today. Tokyo: Syuei publishing 1963. [Book in Japanese]

4. Fujita I, Monoi H, Okudaira N, Ueda M, Ono K, Shimogaki Y, et al. Developing a test of lexical processing in aphasia (TLPA). The Japan Journal of Logopedics and Phoniatrics. 2000;41(2):179-202.[Article in Japanese]
